# Functional MRI (fMRI) Evaluation of Hyperbaric Oxygen Therapy (HBOT) Efficacy in Chronic Cerebral Stroke: A Small Retrospective Consecutive Case Series

**DOI:** 10.3390/ijerph18010190

**Published:** 2020-12-29

**Authors:** Daniela Cevolani, Ferruccio Di Donato, Luigi Santarella, Simone Bertossi, Martino Cellerini

**Affiliations:** 1 Department of Experimental, Diagnostic and Specialty Medicine (DIMES), University of Bologna, c/o Neuroradiology Unit, “Bellaria” Hospital, 40139 Bologna, Italy; 2 Hyperbaric Centre of Bologna, Quarto Inferiore, 40057 Bologna, Italy; ferruccio.didonato@gmail.com (F.D.D.); l.santarella@iperbaricobologna.it (L.S.); simone.bo@gmail.com (S.B.); 3 Neuroradiology Unit, “Bellaria” Hospital, IRCCS Institute of Neurological Sciences, 40139 Bologna, Italy; martino.cellerini@isnb.it

**Keywords:** HyperBaric Oxygen Therapy (HBOT), chronic cerebral stroke, Blood Oxygenation Level-Dependent (BOLD) signal, functional Magnetic Resonance Imaging (fMRI)

## Abstract

**Topics:** Functional Magnetic Resonance Imaging (fMRI) evaluation of HyberBaric Oxygen Therapy (HBOT) effects on chronic cerebral stroke Patients (Pts). **Introduction:** Our aim was to evaluate with fMRI, in a 3 Tesla system, the functional effects of HBOT on the Central Nervous System (CNS) in four Pts with established ischaemic and haemorrhagic cerebral strokes (2 Pts each). To our knowledge, no author used fMRI technique for this purpose, till now. **Methods:** All four Pts underwent a fMRI study before and after 40 HBOT sessions, with a time window of a few days. They carried out two language (text listening, silent word-verb generation) and two motor (hand and foot movements) tasks (30 s On-Off block paradigms). **Results:** After HBOT, all Pts reported a clinical improvement, mostly concerning language fluency and motor paresis. fMRI analysis demonstrated an increase in both the extent and the statistical significance of most of the examined eloquent areas. **Conclusions:** These changes were consistent with the clinical improvement in all Pts, suggesting a possible role of fMRI in revealing neuronal functional correlates of neuronal plasticity and HBOT-related neoangiogenesis. Although only four Pts were examined, fMRI proved to be a sensitive, non-invasive and reliable modality for monitoring neuronal functional changes before and after HBOT.

## 1. Introduction

HyperBaric Oxygen Therapy (HBOT) is a safe technique, successfully used in many pathologies. In our country (Italy), we refer to the European Committee for Hyperbaric Medicine (ECHM), revised in the 2017 Tenth European Committee for Hyperbaric Medicine (10^th^ ECCHM) [1].

Among Type 3 recommendations, chronic stroke is present, with a Level C evidence, where: Type 3, in humans, means “Weak evidence of beneficial action based only on uncontrolled studies (historical control group, cohort study)” and Level C means a “Consensus opinion of experts”.

To our knowledge, no author used fMRI to evaluate HBOT functional effects on the CNS of Pts with established strokes. Our aim was to evaluate functional HBOT effects in chronic stroke Pts’ CNS by fMRI. We chose fMRI because it is a non–invasive technique, allowing the safe and repetitive in vivo study of local functional neuronal changes.

## 2. Materials and Methods

### 2.1. Pts Enrolment and Investigation Protocol

Pts enrolment occurred according to our clinical activity, following the Pts’ requests to improve their clinical status. Since HBOT is allowed for this clinical indication by the 10^th^ ECCHM (even if with weak clinical evidence), Pts were enrolled after an appropriate informed consent and exclusion of any possible contraindications during a thorough clinical examination in the outpatient clinic. In this context, we performed a retrospective study and took into account the last consecutive 4 Pts suffering from chronic cerebral stroke.

### 2.2. Ethical Committee and Written Consent

Our retrospective study was approved by the local Ethical Committee (Comitato Etico di Area Vasta Emilia Centro della Regione Emilia-Romagna, CE-AVEC, Bologna, Italy), Reference Number 258-2020-OSS-AUSLBO. A written informed consent was obtained from each Pt, in accordance with the Declaration of Helsinki.

### 2.3. Patients

- Patient (Pt) 1. Male, 60 years old, right-handed. The patient had a cryptogenetic ischaemic stroke from a left middle cerebral artery (MCA) occlusion 14 months prior to our first visit. Clinically, he presented with a severe, non-fluent aphasia, associated with retained comprehension, dyslexia and dysgraphia, a right upper limb spastic paralysis and a right lower limb paresis. He suffered also from post-stroke depression, mainly due to the constrained absence of social life. At that time, he had just finished both physiotherapy and speech therapy, with minimal results. Speech therapy and physiotherapy were restarted and associated to HBOT during the whole period.
- Pt 2. Male, 68 years old, right-handed. He presented with a chronic multifocal encephalopathy from recurrent cerebral ischaemic strokes 5 years earlier. His clinical examination revealed moderate signs of dysarthria (he sounded clumsy in reading aloud short passages), an unsteady, wide-base gait and dysphagia.
- Pt 3. Male, 48 years old, right-handed. He had a haemorrhagic stroke, involving the right MCA territory 15 years earlier. His past medical history was characterized by recurrent seizures, initially controlled by oxcarbazepine, and ultimately by phenobarbital and carbamazepine. At a clinical examination he presented mild signs of dysarthria (denied by the Pt), upper left limb paralysis, lower left limb paresis, lack of coordination in the right limbs, severe in the lower right extremity and to a lesser extent in the upper right extremity.
- Pt 4. Male, 34 years old, right-handed. The patient suffered from a haemorrhagic transformation of an ischaemic stroke in the left MCA territory 17 months earlier. At a clinical examination he presented a global non fluent aphasia with some deficits in the reading comprehension, a complete spastic hemiplegia of the right upper limb with spastic hypertonia, a partial paresis of the right inferior limb (but he was able to walk with some walking aids) and right hemilateral hypoaesthesia.

At the time of their first outpatient clinic visit all patients were advised to start / resume speech therapy and physiotherapy during HBOT time window, unfortunately with different adhesions (see Results, 3.1. Pts’ Clinical Pictures).

### 2.4. HyperBaric Oxygen Therapy (HBOT)

**Figure 1:**
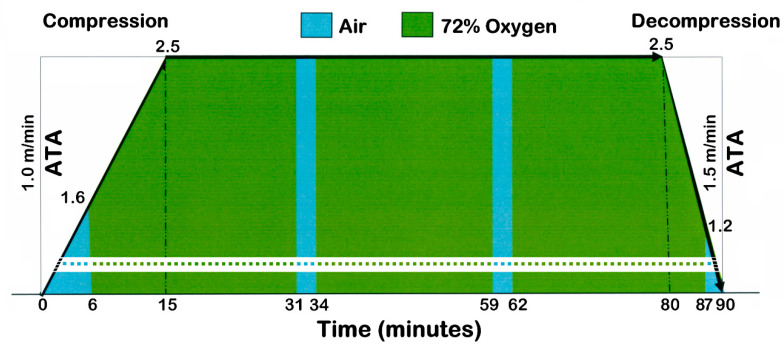
Schematic drawing showing pressure changes in the hyperbaric chamber and alternating respiratory mixtures, during a single 90 min session. Time in minutes on the x axis, Pressure in Absolute Total Ambient pressure (ATA) on the y axis. During the first 15 min, the pressure was slowly increased from 1 ATA to 2.5 ATA with a 0.1 ATA m/min ramp (total rising time 15 min). Once the maximum was reached (2.5 ATA), the pressure was kept constant for 65 min. Then, it was slowly (0.15 ATA/min) decreased down to 1 ATA in a total decreasing time of 10 min. Regarding to respiratory mixtures, patients breathed medical air for the first 6 min (up to the pressure of 1.6 ATA), then began to inhale EAN 72 (Enriched Air Nitrox—72% Oxygen/28% Nitrogen mixture supply) and continued for 3 cycles of 25 min (for a total time of 75 min). Each cycle was interrupted by a 3 min interval of breathing pressurized medical air (shown by the two light blue breaks of 3 min, from 31^st^ to 34^th^ and from 59^th^ to 62^nd^ minute) (Mod. from [3] with permission).

All Pts underwent 40 HBOT sessions, preceded (few days before) and followed (few days after) by Magnetic Resonance (MR) and fMRI examinations. Each HBOT session consisted of a 90 min session in a multiplace air pressurized hyperbaric chamber (Drass Galeazzi, Italy, 1985, capacity 23,800 litres, 12 places, max work pressure 6 Absolute Total Ambient pressure, ATA), at 2.5 ATA, and at 21 ± 2.5 °C temperature. Pts breathed EAN 72 (Enriched Air Nitrox—72% Oxygen/28% Nitrogen), alternated with pressurized medical air, through an oronasal mask, connected to a closed circuit system. This way, we obtained the amount of hyperbaric oxygen corresponding to the breathing of 100% Oxygen at 1.8 ATA, the dosage presumed optimal for the treatment of chronic stroke [2], alternated with pressurized medical air (Figure 1).

All sessions were given on consecutive days, five days a week (except on Sundays and Saturdays), for approximately two months.

### 2.5. MRI Acquisition Parameters

Two consecutive sessions with identical parameters were obtained a few days before and after HBOT.

All data were collected by a 3T Siemens Skyra scanner, equipped by a 64 channel receiver coil. Two 3D isovolumetric morphological and five 2D axial functional sequences were acquired in each Pt. Structural MRIs: sagittal isovolumetric T1 space and T2 space dark fluid (or FLAIR, Fluid Attenuated Inversion Recovery) 1 mm × 1 mm × 1 mm sequences, were acquired in the AC–PC orientation (the standard plane of acquisition in MR, where AC stands for “Anterior Commissure” and PC stands for “Posterior Commissure”), with the following parameters.

T1 space: T1-weighted whole brain (192 slices) sequence with 700 ms repetition time, 11 ms time of echo, 256 × 256 matrix, 1.0 mm slice thickness, 1.0 mm spacing, 0.0 mm gap, 256 × 256 mm^2^ field of view, one excitation, 120° flip angle and 4′44″ total time of acquisition.

T2 space dark fluid: T2-weighted whole brain (176 slices) sequence with 5000 ms repetition time, 428 ms echo time, 1800 ms inversion time, 256 × 256 matrix, 1.0 mm slice thickness, 1.0 mm spacing, 0.0 mm gap, 256 × 256 mm^2^ field of view, one excitation, 120° flip angle and 5′45″ total time of acquisition.

BOLD T2 fMRI: T2*-weighted whole brain (60 axial slices), a single-shot gradient-echo echoplanar (EPI-GE) sequence, with 3000 ms repetition time, 30 ms echo time, 94 × 94 matrix, 3.0 mm slice thickness, 3.0 mm spacing, 0.0 mm gap, 191.9 × 191.9 mm^2^ field of view, one echo, 90° flip angle and 5′15″ total time of acquisition, corresponding to the acquisition of 100 volumes.

### 2.6. fMRI Tasks

In fMRI, it is possible to evoke activations of specific brain areas by using particular “strategies”, often called paradigms [4].

From [4]: … a reliable paradigm should have the following characteristics: (1) The activation induced should be specific. Specificity refers to the ability to localize a function: a high specific paradigm should have a high localizing power, i.e., the ability to select and discover all and only the areas appertaining to that function considered. This way, evoked eloquent areas are defined unambiguously as to anatomical location and extent. (2) The activation induced should be reproducible: evoked eloquent areas have to remain unchanged as to location and extent, through different trials, made in the same and/or in different sessions, thus allowing patient follow-up. (3) The paradigm should be easy to learn by patients having different social and cultural backgrounds. If a patient does not understand clearly what to do or what will happen, he or she obviously will not perform the paradigm correctly and the result will be a suboptimal activation. (4) The paradigm should be short-lasting. The length of time of a paradigm should enable the patient to maintain a high attention level all through the trial; otherwise, again a suboptimal activation will result. Unfortunately, an optimal duration is only a compromise between the time spent by the patient in the magnet and the need to acquire enough data for statistically significant mapping …

When a subject is performing a paradigm (particularly a motor paradigm), there is the possibility he/she moves his/her head excessively. Most software have motion correction procedures, but, if head movements exceed 5 mm, the motion correction is not reliable any more. In these cases, the processing results in motion artefacts and the resulting eloquent areas appear very extended, with irregular borders and often full of gaps.

The most powerful and reliable paradigm is the so-called “block paradigm”. In the case of a typical “block paradigm”, a specific stimulus (an acoustic one or a movement) is maintained for a certain length of time (usually 20–30 s, ON period), followed by absence of stimuli (usually for 30–40 s, OFF period). ON and OFF periods alternate for all the duration of the task (e.g., 5 min), during which a specific BOLD sequence is continuously acquired. After the end of the acquisition (hence off-line), the software takes into account all ON and OFF periods separately, sums each of them, then subtracts the sum value of all OFF periods from the sum value of all ON periods. The resulting differential value (expressed in dimensionless units) is then divided by the whole time of the task. This way, a mean BOLD signal is obtained.

Four “classic” fMRI “block paradigms” tasks were selected from the literature, according to the principles of “the easiest and most reliable” to perform [4,5,6,7,8,9]. Every single task consisted of five consecutive 1 min periods, each one made up of 30 s ON stimulation period followed by 30 s OFF resting periods for a whole task duration of 5 min. The following brain functions were explored:

1. *Receptive language* (passive Test Listening, TL): a 5 min task consisting in the alternation of five 30 s ON periods of listening to one of five complete different 30 s Aesop’s fable stories, each one followed by five 30 s OFF periods of silence.
  It has been shown [4,5,6,7,8] that this paradigm generates eloquent areas in the following two main brain areas:
  - The bilateral Heschl circumvolution (where the primary acoustic area is located) along with the bilateral surrounding so-called “acoustic belt”.
  - Other bilateral eloquent acoustic areas, located in the posterior part of the superior and middle temporal gyrus, often extending unilaterally to the angular and marginal gyri, where the receptive auditory language function is located, the so-called Wernicke’s area. Wernicke’s area is more frequently located in the left hemisphere, where the language function is placed.
2. *Productive language* (covert Words-Verbs Generation, cWVG): a 5 min task consisting in the alternation of five 30 s ON periods of listening to common nouns. Every noun is separated from the following one by 1 s, during which the Pt had to silently think and find the corresponding verb (e.g., bike, to pedal). As the previous task, each of the five 30 s ON period is followed by five 30 s OFF periods of silence.
  It has been shown [4,5,6,7,8] that this paradigm generates eloquent areas in the following brain regions:
  - Bilateral Heschl circumvolutions and respective acoustic belts.
  - Temporal acoustic and Wernicke’s areas.
  - In the frontal operculus, unilaterally, more often on the left side, where the productive language area is located (the so-called Broca’s area).
3. *Left/Right Hand Movement (L/R HM)*: a 5 min task consisting in the alternation of five 30 s ON periods of continuous repetitive opposition of the thumb to each of the four remaining fingers in a sequential tapping (order 2-3-4-5-4-3-2), each one followed by five 30 s OFF periods of rest.
  It has been shown [4,8,9] that this paradigm generates eloquent areas in the following brain regions:
  - The hand primary motor area (contralateral to the side of the hand moved), located in the so-called “central knob” in the precentral gyrus, as predicted by the somatomotor homunculus.
  - The corresponding hand primary sensory area (contralateral to the side of the hand moved) in the post-central gyrus, as predicted by the somatotosensory homunculus. Activation of this area is evoked by muscles and osteotendinous proprioceptors as well as by cutaneous touch receptors, all stimulated by the hand movements.
  - The mirror contralateral primary hand motor area (ipsilateral to the side of the hand moved), less frequent and less extended. This phenomenon is caused by bilateral descending neuronal discharges, due to the mental effort to perform the task correctly, resulting in an unintentional stiffening of the contralateral limb.
  - The supplementary motor area (bilaterally), located antero-medially with respect to primary motor areas. This area comes into action when a complex and coordinate movement has to be carried out.
4. *Left/Right Foot Movement (L/R FM)*: a 5 min task consisting in the alternation of five 30 s ON periods of continuous alternating flexion/extension of the right or the left foot, each one followed by five 30 s OFF periods of rest.
  It has been shown [4,8,9] that this paradigm generates eloquent areas in the following brain regions:
  - In the foot motor area (contralateral to the side of the foot moved), located in the superior and medial part of the precentral gyrus, as by the somatotopic motor homunculus. This area is usually much smaller with respect to the hand’s one, owing to the lesser innervation rate of the inferior limb, accomplishing less fine movements than the hand ones.
  - The corresponding foot primary sensory area (contralateral to the side of the foot moved) in the post-central gyrus, as by the somatotopic sensory homunculus. Also in this case, foot movements activate muscles and osteotendinous proprioceptors as well as cutaneous touch receptors.
  - The supplementary motor area (bilaterally), may be more or less activated, depending on the difficulty of the movement to be carried out. Foot primary and supplementary motor areas are often fused.

### 2.7. fMRI Motor Task Application to Our Pts

After a stroke, some Pts showed motor paralysis in their upper/lower limbs, so they were no more able to perform a motor task. In these cases, we instructed the Pts to think to the same movement.

There are, in fact, some papers showing that to image hand/foot movements, without performing them, was able to evoke eloquent areas in the specific motor areas that would have been activated if the real movement had been performed [10,11]. These eloquent areas are due to the endogenous activation of motor engrams in premotor areas [12].

Based on these considerations, Pt 1 and Pt 4, suffering from right upper limb paralysis, were instructed to think to move their right hand; Pt 3, suffering from left upper limb paralysis, was instructed to move his right uncoordinated hand, the one on the same side of the lesion.

### 2.8. fMRI Data Analysis

Data analysis was performed offline by using the fMRI semiautomatic software package BrainVoyager QX 2.6.1 (Brain Innovation^®^, Maastricht, The Netherlands). All fMRI runs (task by task) were processed individually for every participants. The first 15 s (5 volumes, called dummy volumes), in each fMRI run, were automatically discarded by the 3T system, to allow sequence equilibration and to avoid T1-related relaxation effects. The slice scan time correction, to adjust interleaved acquisition, was automatically carried out by the 3T system. Standard preprocessing was then applied to the functional data. Briefly, the three-dimensional (3D) motion correction was performed by trilinear interpolation and rigid body transformation, using six parameters three translations (X, Y, Z) and three rotations (pitch, roll, and yaw) for realignment: all volumes of the run were spatially aligned to the first one. As to the spatial smoothing of the data, a Gaussian kernel (3D 8 × 8 × 8 FWHM, Full Width Half Maximum) was applied to improve the signal to noise ratio. Single-subject statistical analysis consisted of modelling the active and rest conditions of the block paradigms used within the General Linear Model, via a convolution model and a canonical Haemodynamic Response Function. A 120 s high pass filter was used. Then, functional slices were resampled into 1 mm isotropic voxels by trilinear interpolation and coregistered to the “native” 3D T2 space dark fluid volumes. Finally, functional parametric maps were superimposed to the “native” 3D at the statistical threshold of *p* < 0.005.

## 3. Results

None of the Pts showed any complications during and following HBOT. It was a necessary prerequisite. If HBOT had any serious side effects, it would not have been possible to use it.

### 3.1. Pt’s Clinical Pictures

- Pt 1 resumed both speech therapy and motor rehabilitation during the period of HBOT. After HBOT, aphasia improved noticeably. The recovery of the ability to speak allowed Pt 1 to regain some social life. Accordingly, the negative attitude was replaced by positivity, good mood and interest. Right lower limb paresis improved with better gait and stance. However, the right hand paralysis did not improve, except for a decrease in spasticity.
- Pt 2 did not undergo physiotherapy nor speech therapy. After HBOT, Pt 2 showed a marked clinical improvement in the language fluency, such that he was able to read aloud a written text, without a hitch. Ataxia and dysphagia improved as well.
- Pt 3 refused speech therapy and rehabilitation, but decided autonomously to practice a sport (swimming). After HBOT, he dramatically improved his speech fluency. The left lower limb paresis improved together with an increase in walking autonomy, but he was still unable to flex and extend the left foot rhythmically. Also the right limbs coordination improved, allowing the Pt to perform the requested upper limb motor task correctly. On the contrary, the left upper limb paralysis did not change at all.
- Pt 4. Even without any speech therapy or rehabilitation, after HBOT, Pt4 showed a good improvement in the common everyday acts of his life, as speaking and understanding and also in walking, but not in his upper limb paralysis.

### 3.2. Lesion Morphology Before HBOT

**Figure 2:**
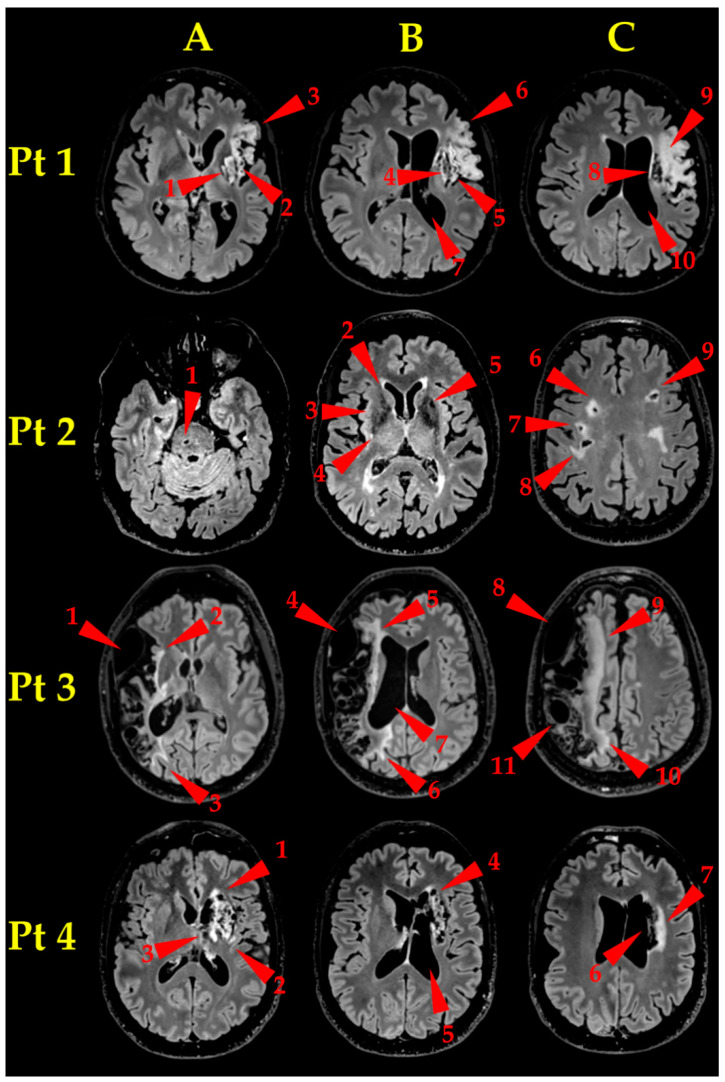
Lesion Morphology before HBOT. FLAIR T2-weighted images. Three axial slices at different levels (A, B, C) are shown for each Pt. Red arrowheads point to the lesions. **Pt 1**: capsulo-lenticular cavitation (A, B, arrowhead 1 and 4, respectively), insular degeneration (A, B, arrowheads 2 and 5, respectively), *corona radiata* cavitation (C, arrowhead 8), malacic *frontal operculum* (A, B, arrowheads 3 and 6, respectively) and fronto-lateral cortex (C, arrowhead 9), ex-vacuum enlargement of the adjacent segment of left lateral ventricle (B, C, arrowheads 7 and 10, respectively). **Pt2:** gliotic areas in subcortical and para-ventricular deep white substance, bilaterally, but prevailing on the right side (C, arrowheads 6–9), extending downwards to the basal ganglia (B, arrowheads 2–5) and to the right pons (A, arrowhead 1). **Pt 3**: wide fronto-parieto-temporal malacic area (whose medial edge is delimited by arrowheads 2–3, 5–6 and 9–10, in A, B and C, respectively). *Ex vacuo* enlargement of right lateral ventricle (B, arrowhead 7). Very extended cyst (A, B, C, arrowheads 1, 4 and 8, respectively) and smaller cyst (C, arrowhead 11). **Pt 4**: gliotic-malacic area in the left capsulo-lenticular nucleus, partially involving the *insula* (A, arrowhead 1–3), extending to the left caudate nucleus (B, arrowhead 4) and to the ipsilateral corona radiata (C, arrowheads 6, 7). Note also the moderate dilation of the left lateral ventricle (B, arrowhead 5).

- Pt 1. Brain MR examination (Figure 2, Pt 1, A, B, C) showed an established ischaemic lesion in the deep and superficial territory of the left MCA. The lesion appeared as a wide malacic area (hypointense on T1-weighted, unhomogeneously hyperintense on T2-weighted FLAIR sequences) and showed signs of previous superficial haematic staining. A capsulo-lenticular cavitation (A and B, arrowheads 1 and 4, respectively) is present, with concomitant Wallerian degeneration of the bulb, extending laterally to the *insula* (A and B, arrowheads 2 and 5, respectively) and upward to the left *corona radiata* (C, arrowhead 8), which appeared also cavitated. The malacic area extends to the left frontal operculum (A and B, arrowheads 3 and 6, respectively), where the Broca’s area is located, and upward to the left fronto-lateral cortex (C, arrowhead 9). Finally, the massive tissue destruction caused an ex-vacuum enlargement of the adjacent segment of left lateral ventricle (B, C, arrowheads 7 and 10, respectively).
- Pt 2. Brain MR examination (Figure 2, Pt 2, A, B, C) showed many bilateral gliotic areas, prevalent on the right side, located mainly in the bi-hemispherical subcortical and para-ventricular deep white matter (C, arrowheads 6–9), extending downwards to the basal ganglia (B, arrowheads 2–5) and to the right side of the pons (A, arrowhead 1). They appeared as many small areas, hyperintense on T2-weighted images, consistent with chronic small vessels disease.
- Pt 3. Brain MR examination (Figure 2, Pt 3, A, B, C) showed a very wide right fronto-parieto-temporal malacic area (whose medial border is pointed to by arrowheads 2–3, 5–6 and 9–10, in A, B and C, respectively), involving the superficial territories of the right MCA, with *ex-vacuo* enlargement of the corresponding right lateral ventricle (B, arrowhead 7). It is also clearly visible a bulky arachnoid cyst, extending from the temporal pole to the anterior Sylvian region (A, B and C, arrowheads 1, 4 and 8, respectively) and a relatively smaller one, located in the parietal region (C, arrowhead 11).
- Pt 4. Brain MR examination (Figure 2, Pt 4, A, B, C) showed a gliotic-malacic area in the left capsulo-lenticular nucleus, partially involving the *insula* (A, arrowhead 1–3), extending upwards to the left caudate nucleus (B, arrowhead 4) and to the ipsilateral *corona radiata* (C, arrowhead 6, 7). Finally, the left lateral ventricle showed a moderate dilatation (C, arrowhead 5).

All the aforementioned lesions showed no visible macroscopic morphological changes in all Pts after HBOT (Supplementary Figure S1).

### 3.3. MRI

To facilitate the Figure’s comprehension, it should be noted that, according to the radiological convention of tomographic imaging, the right side is imaged on the left side and *vice versa*, as well as that motor pathways are crossed (the right hemisphere commands the movements of the left limbs of the body and *vice versa*). The software analysis releases colour parametric maps, whose colour and extension may change depending on statistical significance. The yellow-white colour on the map corresponds to higher statistical significance, whereas red-orange colour to less statistical significance.

#### 3.3.1. Language

**Figure 3:**
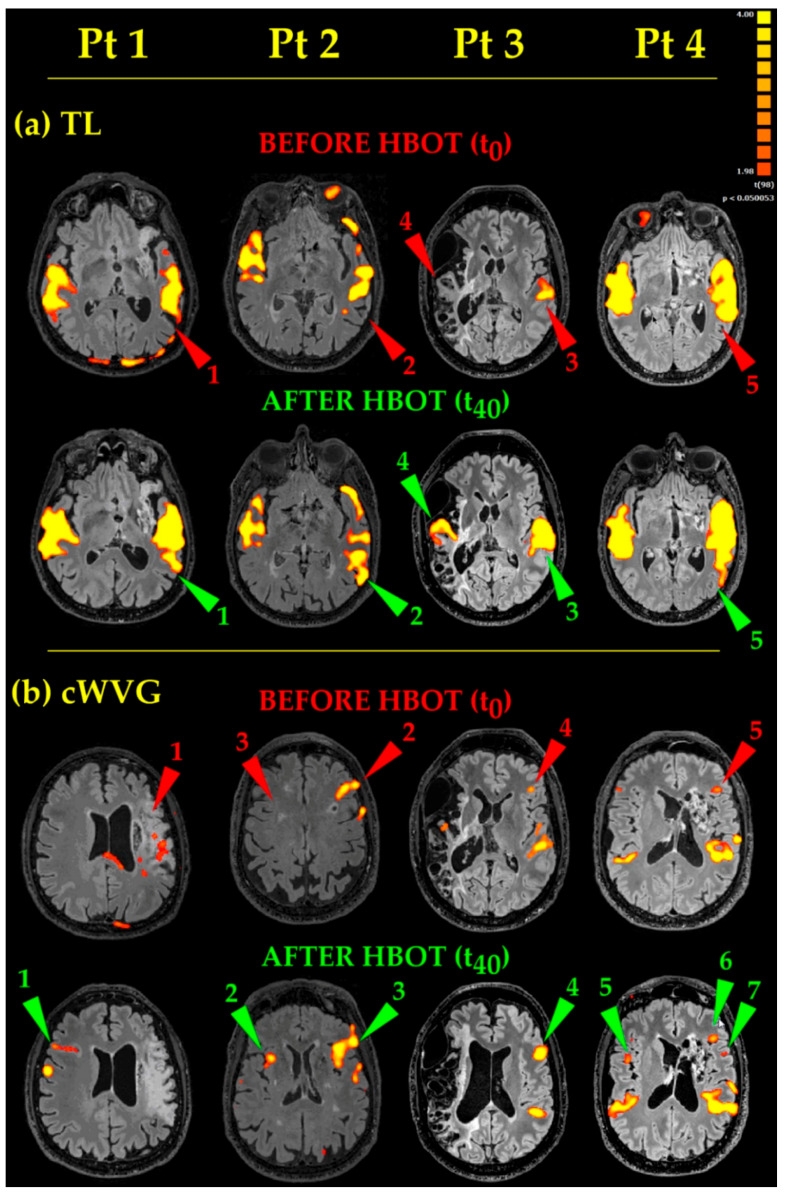
Language eloquent areas. Functional maps few days before (T_0_) and few days after 40 (T_40_) HBOT 90 min sessions, in the four Pts examined. (**a**) TL, Text Listening task; (**b**) cWVG, covert Word-Verb Generation task. On the upper right corner, the colour scale of the statistical parametric maps at the statistical significance of *p* < 0.005. Eloquent areas description in the text.

- As to *Receptive Language* (Figure 3, TL), the acoustic tasks evoked bilateral (except for Pt 3) temporal eloquent areas, located in the superior and middle temporal gyri (Figure 3, TL, Before HBOT, Pts 1, 2, 4, red arrowheads 1, 2, 5, respectively). After HBOT, the same acoustic tasks evoked temporal eloquent areas looking more extended antero-posteriorly and superiorly (involving the presumed Wernicke’s area) up to include the left angular gyrus too, in almost all Pts (Figure 3, TL, Pts 1, 2, 4, cp. red 1, 2, 5, Before HBOT, and green 1, 2, 5, After HBOT, arrowheads). In Pt 3, whose brain was one of the most damaged at the temporal lobe level, before HBOT, TL task generated only a unilateral, left sided, temporal eloquent acoustic area (Figure 3, TL, Before HBOT, Pt 3, red arrowhead 3). After HBOT, Pt 3′s eloquent areas became bilateral, with the appearance of a new activated area on the contralateral side (compare Figure 3, TL, After HBOT, Pt3, green arrowhead 4 with Figure 3, TL, Before HBOT, Pt 3, red arrowhead 4).

- *Productive Language* tasks (Figure 3, cWVG). Before HBOT, the eloquent areas in the left frontal operculum (where the Broca’s area, the structure at the basis of language fluency, is located) appeared small and barely visible in Pts 3 and 4 (Figure 3, cWVG, Before HBOT, Pts 3 and 4, red arrowheads 4 and 5, respectively). After HBOT, the same cWVG task evocated more extended and more significant (more yellow than orange-red) activated areas in Pts 2, 3 and 4 (Figure 3, cWVG, Pts 2, 3 and 4, compare red 2, 4, 5 and green 3, 4, 6 arrowheads, Before and After HBOT, respectively). A second small fronto-opercular activation appeared in Pt4 (note the green arrowhead 7 in Pt 4 in Figure 3, cWVG, After HBOT). In Pt 1, having a massive damage in the left hemisphere, no eloquent fronto-opercular areas appeared before HBOT (Figure 3, cWVG, Before HBOT, Pt1, red arrowhead 1). After HBOT, a clear-cut new frontal opercular eloquent area appeared on the contralateral right side (Figure 3, cWVG, After HBOT, Pt 1, green arrowhead 1), in a nearly mirror position (coherent with a new contralateral Broca’s area). Also in Pt 2 and Pt 4 a new eloquent area appeared on the right side, in between frontal operculum and insula (Figure 3, cWVG, After HBOT, Pt 2 and Pt 4, green arrowheads 2 and 5, respectively). Finally, the acoustic cWVG stimuli evoked the expected bilateral (except for Pt3) temporal Heschl’s and Wernicke’s activations, which were more extended and more statistically significant in all Pts, after HBOT).

#### 3.3.2. Movements

**Figure 4:**
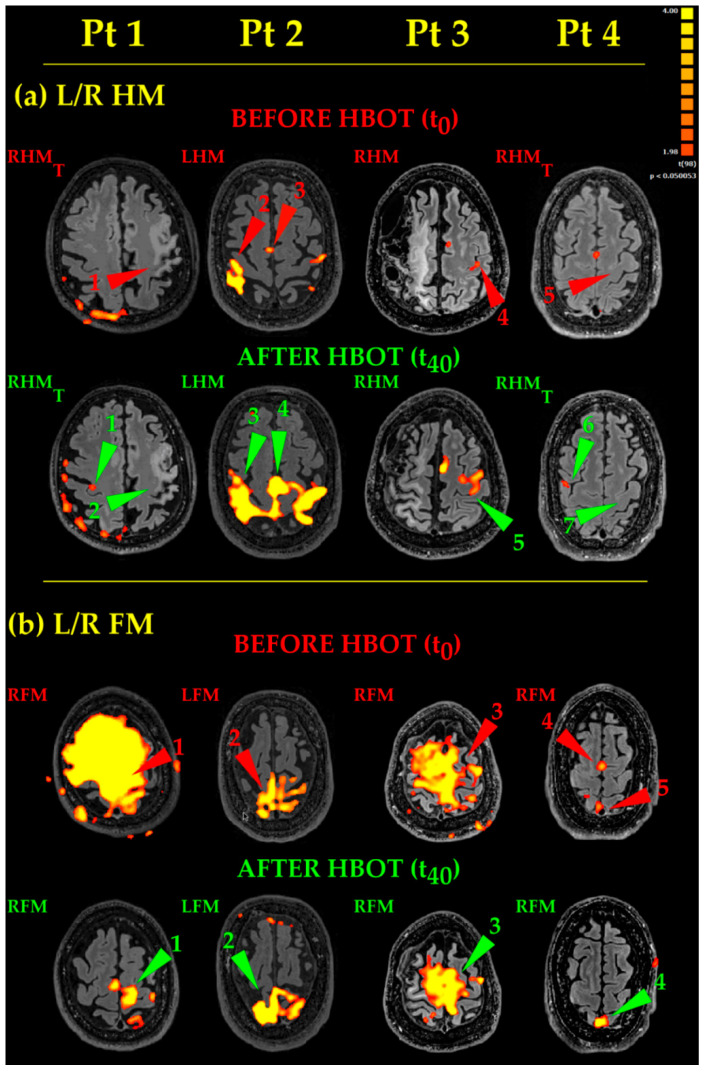
Motor eloquent areas. Functional maps few days before (T_0_) and few days after 40 (T_40_) HBOT 90 min sessions, in the four Pts examined. (**a**) L/R HM, Left/Right Hand Movements. LHM, left hand movement; RHM, right hand movement; RHM_T_, right hand movement thought. (**b**) L/R FM, Left/Right Foot Movements. LFM, left foot movement, RFM, right foot movement. On the upper right corner, the colour scale of the statistical parametric maps at the statistical significance of *p* < 0.005. Eloquent areas description in the text.

- *Hand Movements* (Figure 4, L/R HM). Pt 1 and Pt 4 thought to the movement of their right hand, but no activation occurred in the classical hand motor area (central omega/epsilon) (Figure 4, L/R HM, Before HBOT, Pt 1 and Pt 4, RHM_T_, red arrowheads 1 and 5, respectively). After HBOT, the same thinking to RHM did not evoke again any activations in the expected left hand motor areas (Figure 4, L/R HM, After HBOT, Pt1 and 4, RHM_T_, green arrowheads 2 and 7). It evoked instead a small, dot-like activation in the “wrong” right motor cortex only (Figure 4, L/R HM, After HBOT, Pt 1 and Pt 4, RHM_T_, green arrowheads 1 and 6). Likewise, Pt 3 moved his right uncoordinated upper limb, generating a very small activation before HBOT (Figure 4, L/R HM, Before HBOT, Pt3, RHM, red arrowhead 3). After HBOT, Pt 3 accomplished a near normal RHM (Figure 4, L/R HM, After HBOT, Pt3, RHM, green arrowhead 5). Pt 2 was the least compromised in his motor functions and performed his RHM almost normally before HBOT (Figure 4, L/R HM, Before HBOT, Pt2, RHM, red arrowhead 2). After HBOT, eloquent areas become much more extended, bilateral and more significant (Figure 4, L/R HM, After HBOT, Pt 2, RHM, green arrowhead 3), together with the enlargement of the supplementary motor area (compare red arrowhead 3 with green arrowhead 4, respectively, in Figure 4, L/R HM, Pt 2, Before and After HBOT). Activated areas were very extended: this because Pt 2 used excessive energy in performing his task.
- *Foot Movements* (Figure 4, L/R FM). As to Pt 1 and Pt 3, the huge yellow activated areas (of lesser extent in Pt 3) are coherent with very large, uncoordinated movements, occurring during the unsuccessful attempts to execute properly the RFM task before HBOT (Figure 4, L/R FM, Before HBOT, Pt 1 and Pt 3, RFM, red arrowheads 1 and 3, respectively). After HBOT, Pt 1 accomplished RFM task more easily and with more coordination, giving rise to an almost normal activation (Figure 4, L/R FM, After HBOT, Pt 1, RFM, green arrowhead 1). On the other hand, Pt 3, while showing some improvement, did not succeeded in performing his RFM task properly; RFM eloquent areas decreased a bit, while remaining more extended than normal (Figure 4, L/R FM, After HBOT, Pt 3, RFM, green arrowhead 3). In Pt2, the LFM before HBOT showed an irregular fragmented map (Figure 4, L/R FM, Before HBOT, Pt 2, LFM, red arrowhead 2); after HBOT, Pt 2 greatly improved the execution of his task, showing more significant and better defined eloquent areas (Figure 4, L/R FM, After HBOT, Pt 2, LFM, green arrowhead 2). Finally, in Pt 4, RFM resulted in a very small activation before HBOT (Figure 4, L/R FM, Before HBOT, Pt 4, RFM, red arrowhead 5) and in an activated supplementary area, due to his coordination effort to complete the task (Figure 4, L/R FM, Before HBOT, Pt 4, RFM, red arrowhead 4). After HBOT, actually, he succeeded in performing his RFM more smoothly and with less effort, showing, as a result, a bit more extended, more significant eloquent area (Figure 4, L/R FM, After HBOT, Pt 4, RFM, green arrowhead 4).

## 4. Discussion

### 4.1. HBOT Recommendation in Chronic Stroke Pts

In the 2017 10^th^ ECCHM [1] there was a consensus agreement in recommending HBOT for Pts with chronic stroke (Table 2, p. 27, Recommendations on the indications accepted for HBOT, Type 3).

The ECHM document is from 2016. In the meantime, many papers [13,14,15,16,17,18,19,20,21,22,23,24,25] have been published in favour of HBOT, so that its level of evidence may have been increased.

Our preliminary results confirm the effectiveness of HBOT in improving the clinic of cerebral stroke Pts [24,26]. We believe that the association of HBOT with rehabilitation physiotherapy and speech therapy is the most effective way to improve neurological symptoms [20]. On the basis of this conviction, we advised all Pts to follow this procedure. Actually, Pt1, who was the only one who faithfully followed our advice, was also the one who showed the greatest improvement. This improvement apparently occurs without macroscopic changes in brain morphology.

### 4.2. HBOT Brain Effects And Mechanisms

Overall, HBOT therapy resulted in an increase of the extension and/or statistical significance of most of the evoked eloquent areas in all Pts. It should be emphasized that the observed changes strictly correlated with the Pts’ clinical improvements. In Pts with such a severe incoordination to hinder the correct performance of the task before HBOT, the clinical improvement after HBOT was coherent with both the decrease in motion artefacts and the increased specificity in the resulting activated maps. In some cases, new eloquent areas appeared (Figure 3, After HBOT, TL Pt3, and cWVG, Pt 1, Pt 2 and Pt 4, green arrowheads 1, 2 and 5, respectively).

Some papers [24,27,28] reported that HBOT is able to stimulate neuroplasticity even years after a brain insult.

Although the mechanisms underlying HBOT brain effects have to be clearly defined yet [28], it seems that many factors are involved: inflammation modulation [25], strengthening of the antioxidant endogenous system [29,30], neoangiogenesis stimulation in the damaged tissues [21,31] and tissue repair stimulation by bone marrow staminal cells mobilization [32]. All these mechanisms could account for HBOT ability to stimulate neoangiogenesis and neuroplasticity even years after the brain insult.

### 4.3. Pressure Values in the Hyperbaric Chamber

We choose breathing EAN 72 (Enriched Air Nitrox—72% Oxygen / 28% Nitrogen) at 2.5 ATA, to obtain the amount of hyperbaric oxygen corresponding to the breathing of 100% Oxygen at 1.8 ATA, the dosage presumed optimal for the treatment of chronic stroke [2].

The intermittent supply of the therapeutic gas mixture was chosen in order to reduce the likelihood of the appearance of oxygen neurotoxicity [33].

Finally, we choose to use an enriched mixture in a higher absolute environmental pressure than would have been necessary if 100% Oxygen had been used. This also allowed treating simultaneously, in the same chamber, many Pts having other pathologies and requiring a higher oxygen dose.

### 4.4. fMRI

To our knowledge, this is the first study employing fMRI to evaluate HBOT CNS effects, in Pts with chronic stroke. In our small consecutive series, fMRI has proved to be a very sensitive, non-invasive and reliable tool for monitoring brain functional changes, and therefore evaluate the functional effects before and after HBOT.

## 5. Conclusions

fMRI is a non invasive, robust and reliable technique to assess and monitor neuronal functional changes. Although limited by the small number of cases, our work discloses a new possible future role of fMRI to evaluate the effects of HBOT in Pts with chronic stroke. On the basis of this experience, we are planning a future investigational study.

## Data Availability

The data presented in this study are available on request from the corresponding author.

## Supplementary Materials

The following is available online at https://www.mdpi.com/1660-4601/18/1/190/s1, Figure S1: Lesions Morphology after HBOT.

## References

[1] Mathieu D., Marroni A., Kot J. (2017). Tenth European Consensus Conference on Hyperbaric Medicine: Recommendations for and non-accepted clinical indications and practice of hyperbaric oxygen treatment. Diving Hyperb. Med..

[2] Marroni A. (1988). Hyperbaric Oxygen and In-Water Rehabilitation in Complete Stroke. J. Hyperb. Med..

[3] Di Donato F. (2017). L’orecchio in Immersione.

[4] Cevolani D., Agati R., Leonardi M., Scarabino T., Pollice S., Propolizio T. (2017). Use of fMRI activation paradigms: A presurgical tool for mapping brain function. High Field Brain MRI. Use in Clinical Practice.

[5] FitzGerald D.B., Cosgrove G.R., Ronner S., Jiang H., Buchbinder B.R., Belliveau J.W., Rosen B.R., Benson R.R. (1997). Location of language in the cortex: A comparison between functional MR imaging and electrocortical stimulation. AJNR Am. J. Neuroradiol..

[6] Gaillard W.D., Balsamo L., Xu B., McKinney C., Papero P.H., Weinstein S., Conry J., Pearl P.L., Sachs B., Sato S. (2004). fMRI language task panel improves determination of language dominance. Neurology.

[7] Holland S.K., Plante E., Weber Byars A., Strawsburg R.H., Schmithorst V.J., Ball W.S. (2001). Normal fMRI brain activation patterns in children performing a verb generation task. NeuroImage.

[8] Sunaert S. (2006). Presurgical planning for tumor resectioning. J. Magn. Reson. Imaging.

[9] Kim S.G., Ashe J., Georgopoulos A.P., Merkle H., Ellermann J.M., Menon R.S., Ogawa S., Ugurbil K. (1993). Functional imaging of human motor cortex at high magnetic field. J. Neurophysiol..

[10] Naito E., Kochiyama T., Kitada R., Nakamura S., Matsumura M., Yonekura Y., Sadato N. (2002). Internally simulated movement sensations during motor imagery activate cortical motor areas and the cerebellum. J. Neurosci..

[11] Yuan H., Liu T., Szarkowski R., Rios C., Ashe J., He B. (2010). Negative covariation between task-related responses in alpha/beta-band activity and BOLD in human sensorimotor cortex: An EEG and fMRI study of motor imagery and movements. NeuroImage.

[12] Hermes D., Vansteensel M.J., Albers A.M., Bleichner M.G., Benedictus M.R., Mendez Orellana C., Aarnoutse E.J., Ramsey N.F. (2011). Functional MRI-based identification of brain areas involved in motor imagery for implantable brain–computer interfaces. J. Neural Eng..

[13] Zhai W.V., Sun L., Yu Z.Q., Chen G. (2016). Hyperbaric oxygen therapy in experimental and clinical stroke. Med. Gas Res..

[14] Hu Q., Manaenko A., Bian H., Guo Z., Huang J.L., Guo Z.N., Yang P., Tang J., Zhang J.H. (2017). Hyperbaric oxygen reduces infarction volume and hemorrhagic transformation through ATP/NAD+/Sirt1 pathway in hyperglycemic middle cerebral artery occlusion rats. Stroke.

[15] Ostrowski R.P., Stępień K., Pucko E., Matyja E. (2017). The efficacy of hyperbaric oxygen in hemorrhagic stroke: Experimental and clinical implications. Arch. Med. Sci..

[16] Tal S., Hadanny A., Sasson E., Suzin G., Efrati S. (2017). Hyperbaric Oxygen Therapy can induce angiogenesis and regeneration of nerve fibers in traumatic brain injury patients. Front. Hum. Neurosci..

[17] Liska G.M., Lippert T., Russo E., Nieves N., Borlongan C.V. (2018). A dual role for hyperbaric oxygen in stroke neuroprotection: Preconditioning of the brain and stem cells. Cond. Med..

[18] Gonzales-Portillo B., Lippert T., Nguyen H., Lee J.Y., Borlongan C.V. (2019). Hyperbaric oxygen therapy: A new look on treating stroke and traumatic brain injury. Brain Circ..

[19] Golan H., Makogon B., Volkov O., Smolyakov Y., Hadanny A., Efrati S. (2020). Imaging-based predictors for hyperbaric oxygen therapy outcome in post-stroke patients. Report 1. Med. Hypotheses.

[20] Schiavo S., Richardson D., Santa Mina D., Buryk-Iggers S., Uehling J., Carroll J., Clarke H., Djaiani C., Gershinsky M., Katznelson R. (2020). Hyperbaric oxygen and focused rehabilitation program: A feasibility study in improving upper limb motor function after stroke. Appl. Physiol. Nutr. Metab..

[21] Wang Y., Gao Y., Lu M., Liu Y. (2020). Long-term functional prognosis of patients with aneurysmal subarachnoid hemorrhage treated with rehabilitation combined with hyperbaric oxygen. Case-series study. Medicine.

[22] Zhong X., Shan A., Xu J., Liang J., Long Y., Du B. (2020). Hyperbaric oxygen for severe traumatic brain injury: A randomized trial. J. Int. Med. Res..

[23] Hadanny A., Rittblat M., Bitterman M., May-Raz I., Suzin G., Boussi-Gross R., Zemel Y., Bechor Y., Catalogna M., Efrati S. (2020). Hyperbaric oxygen therapy improves neurocognitive functions of post-stroke patients—A retrospective analysis. Restor. Neurol. Neurosci..

[24] Mozayeni B.R., Duncan W., Zant E., Love T.L., Beckman R.L., Stoller K.P. (2019). The National Brain Injury, Rescue and Rehabilitation Study—A multicenter observational study of hyperbaric oxygen for mild traumatic brain injury with post-concussive symptoms. Med. Gas Res..

[25] Liang F., Sun L., Yang J., Liu X.H., Zhang J., Zhu W.Q., Yang L., Nan D. (2020). The effect of different atmosphere absolute hyperbaric oxygen on the expression of extracellular histones after traumatic brain injury in rats. Cell Stress Chaperones.

[26] Boussi-Gross R., Golan H., Volkov O., Bechor Y., Hoofien D., Beeri M.S., Ben-Jacob H., Efrati S. (2015). Improvement of memory impairments in poststroke patients by hyperbaric oxygen therapy. Neuropsychology.

[27] Efrati S., Fishlev G., Bechor Y., Volkov O., Bergan J., Kliakhandler K., Kamiager I., Gal N., Friedman M., Ben-Jacob E. (2013). Hyperbaric oxygen induces late neuroplasticity in post stroke patients—Randomized, prospective trial. PLoS ONE.

[28] Rosario E.R., Kaplan S.E., Khonsari S., Vazquez G., Solanki N., Lane M., Brownell H., Rosenberg S.S. (2018). The effect of hyperbaric oxygen therapy on functional impairments caused by ischemic stroke. Neurol. Res. Int..

[29] Thom S.R. (2011). Hyperbaric oxygen: Its mechanisms and efficacy. Plast. Reconstr. Surg..

[30] Alleva R., Di Donato F., Strafella E., Staffolani S., Nocchi L., Borghi B., Pignotti E., Santarelli L., Tomasetti M. (2011). Effect of ascorbic acid-rich diet on in vivo-induced oxidative stress. Br. J. Nutr..

[31] Sheikh A.Y., Gibson J.J., Rollins M.D., Hopf H.W., Hussain Z., Hunt T.K. (2000). Effect of Hyperoxia on Vascular Endothelial Growth Factor Levels in a Wound Model. Arch. Surg..

[32] Goldstein L.J., Gallagher K.A., Bauer S.M., Bauer R.J., Baireddy V., Liu Z.J., Buerk D.G., Thom S.R., Velazquez O.C. (2006). Stem Cell mobilization by hyperbaric oxygen. Am. J. Physiol. Heart Circ. Physiol..

[33] Hamilton R.W. (1997). Tolerating oxygen exposure. SPUMS J..

