# Supplementary material for: Functional MRI (fMRI) Evaluation of Hyperbaric Oxygen Therapy (HBOT) Efficacy in Chronic Cerebral Stroke: A Small Retrospective Consecutive Case Series
Source: Int J Environ Res Public Health. 2020 Dec 29;18(1):190. doi: 10.3390/ijerph18010190 (PMC7794810; doi:10.3390/ijerph18010190)
Supplement: Supplementary file 1 [file ijerph-18-00190-s001.pdf]

International Journal of
Environmental Research and Public Health

MDPI

Supplementary Materials

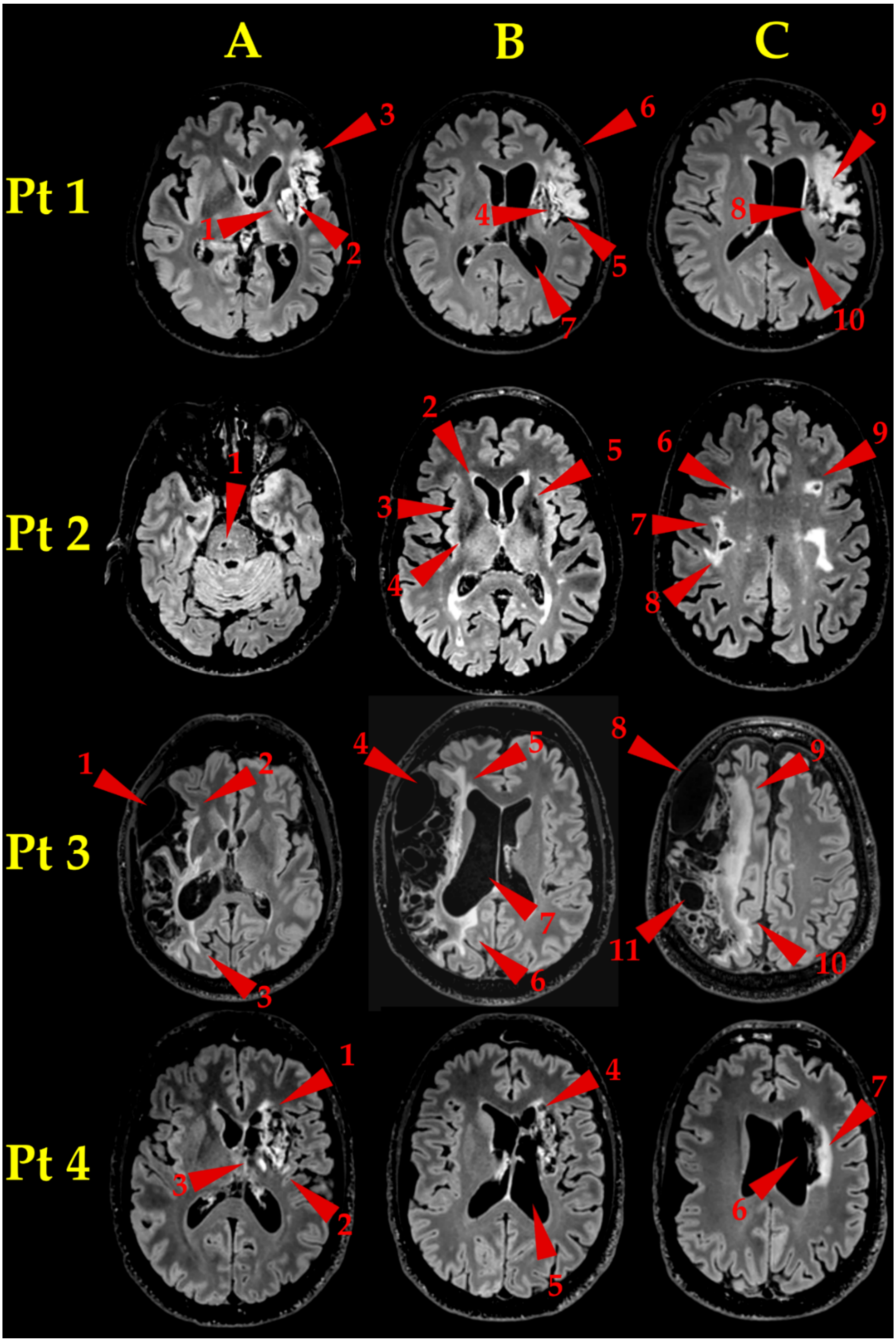

Figure S1. Lesions Morphology after HBOT. FLAIR T2-weighted images. Three axial slices at different levels (A, B, C) are shown for each Pt. Red arrowheads point to the lesions. Legends and arrowheads numbers as in Figure 1. Note some slight differences in Pts' positioning: actually, it is impossible to place the head in exactly the same position in different sessions. In all Pts, brain morphology showed no visible macroscopic changes after HBOT.
